# A noninferiority trial comparing left paratracheal pressure versus cricoid pressure on tracheal intubation conditions using the Pentax Airway Scope

**DOI:** 10.1038/s41598-022-20681-y

**Published:** 2022-09-28

**Authors:** Ha Yeon Kim, Jee Hwan Moon, Hee Yeon Park, Sang Kee Min, Jong Yeop Kim

**Affiliations:** 1 Department of Anesthesiology and Pain Medicine, Ajou University School of Medicine, Suwon, Korea; 2 Department of Anesthesiology and Pain Medicine, Gachon University, Gil Medical Center, Incheon, Korea

**Keywords:** Anatomy, Medical research

## Abstract

There are no studies evaluating the effect of left paratracheal pressure (PP) on difficulty of intubation using Pentax Airway Scope (Pentax), so we conducted this study to show that PP was not inferior to cricoid pressure (CP) in intubation time using the Pentax. Patients aged 19–70 years, with an American Society of Anesthesiologists physical status 1 or 2, and undergoing otorhinolaryngological, gynecological surgery, and cholecystectomy at a single university hospital were enrolled. Intubation was performed using the Pentax under PP or CP of 30 N. The primary outcome was intubation time, and the secondary outcomes were tube advancement difficulty and glottis view. The mean difference in intubation time (PP group − CP group) was − 4.19 s (95% CI − 8.24 to − 0.15), which was within the non-inferiority margin of 6.5 s, thus proving non-inferiority of the PP compared with the CP group. However, the score for tube advancement difficulty was significantly lower in the PP group than in the CP group (p = 0.02). PP did not prolong the intubation time and showed comparable intubation conditions to CP in intubation using the Pentax. Therefore, PP may be a good alternative maneuver to prevent gastric regurgitation during intubation using the Pentax.

## Introduction

In 1961, Sellick proposed a maneuver to prevent regurgitation of gastric contents during anesthetic induction^1^. This maneuver induced a transient esophageal occlusion by pressing the cricoid cartilage backwards. Such cricoid pressure (CP) has been widely used in patients at high risk for regurgitation of gastric contents. However, several studies have raised questions about the efficacy of CP in preventing gastric regurgitation^2–4^. In addition, there were safety issues regarding CP, such as difficult visualization of the vocal cords^5^, airway obstruction^6^, difficulty with manual ventilation^7^, paradoxical opening of the lower esophageal sphincter^8^, and esophageal injury^9^.

Pentax Airway Scope (Pentax; Hoya Co., Tokyo, Japan) is one of the most widely used videolaryngoscopes, allowing visualization of the glottis without alignment of the oro-pharyngeal–trachea axes. Pentax has shown increased usefulness in both emergency and difficult tracheal intubations compared with direct laryngoscopy or use of other videolaryngoscopes^10,11^. However, the application of CP in intubation using Pentax may have negative effects, including difficulty of tube advancement and prolonged intubation time^12,13^. Therefore, other preventive strategies are needed when using Pentax in patients at risk of gastric regurgitation.

Recently, left paratracheal pressure (PP), which involves pressure on the space between the trachea and the sternocleidomastoid muscle just above the clavicle, emerged as an alternative method for preventing gastric regurgitation. A previous study showed that left PP compressed the esophagus as effectively as CP^14^. To date, there are no studies evaluating the effect of PP on difficulty of intubation using Pentax. We hypothesized that PP does not worsen intubation conditions when compared with CP during intubation using Pentax. The aim of this study was to show that PP was not inferior to CP in terms of intubation time and conditions during intubation using Pentax.

## Results

**Figure 1:**
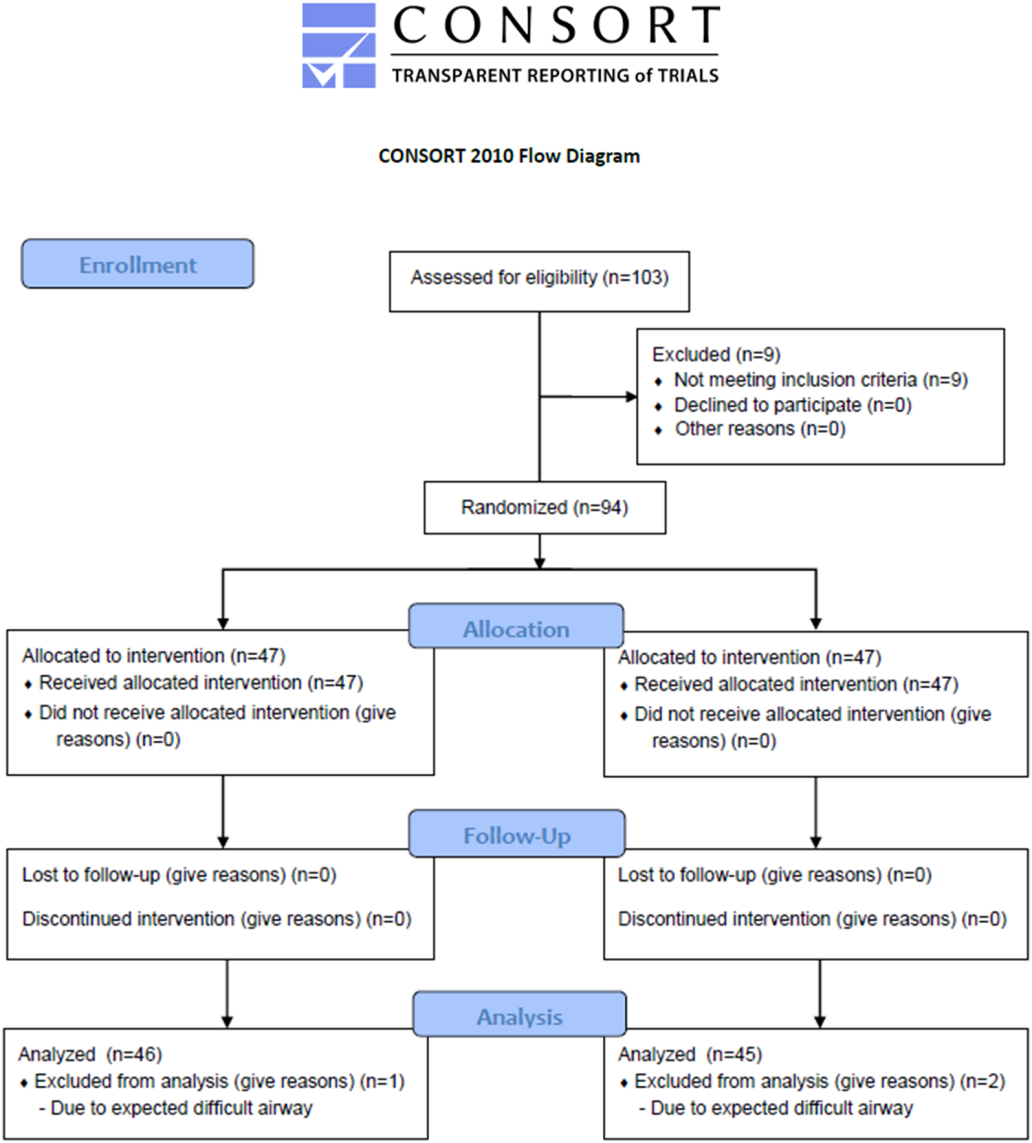
The CONSORT flow chart.

Of 103 patients, nine were excluded, seven because of loose teeth and two because of high body mass index > 35 kg/m^2^. Ninety-four patients were enrolled without follow-up loss from December 2019 to April 2020. They were randomized into two groups with 47 patients per group. However, three patients (one patient in the PP group and two patients in the CP group) were further excluded due to Mallampati grade 4. Finally, 91 patients (46 patients in the PP group and 45 patients in the CP group) were analyzed. The measurement of the AP diameter of the esophagus was performed in only 77 patients (39 patients in the PP group and 38 patients in the CP group) due to the problem of ultrasound accessibility. Other outcome measurements were collected from all patients. The CONSORT flow chart is shown in Fig. 1.

Patients’ demographics and details related to surgery were comparable between the two groups (Table 1). The esophagus was identified in all 77 patients who were examined by ultrasound. The mean AP diameters of the esophagus were significantly decreased after applying PP using the ultrasound probe (axial: 7.8 ± 1.1 cm vs. 5.4 ± 1.2 cm, *p* < 0.001; sagittal: 6.9 ± 1.7 cm vs. 4.6 ± 0.7 cm, *p* < 0.001; before vs. after PP, respectively).

**Table 1:** Patients’ demographics and details related to surgery.

	PP group (n = 46)	CP group (n = 45)	*P*-value
Sex, male n (%)	15 (33)	15 (33)	0.94
Age, years	42.6 ± 10.9	42.5 ± 11.6	0.98
Weight, kg	67.8 ± 15.4	68.8 ± 13.4	0.75
Height, cm	164.1 ± 8.5	166.0 ± 9.4	0.32
Body mass index, kg/m^2^	25.0 ± 4.0	24.9 ± 4.1	0.95
ASA classification I/II, n	32/14	36/9	0.25
**Type of surgery, n (%)**			0.56
Otorhinolaryngology surgery	17 (37)	18 (40)	
Gynecological surgery	12 (26)	12 (27)	
Cholecystectomy	17 (37)	15 (33)	
Operation time, min	48 (35–83)	40 (30–78)	0.19
Anesthesia time, min	83 (70–129)	75 (60–120)	0.10

*ASA* American society of anesthesiologists.

Continuous variables are presented as mean ± SD or median (25th to 75th quartile). Nominal variables are presented number (frequency).

**Figure 2:**
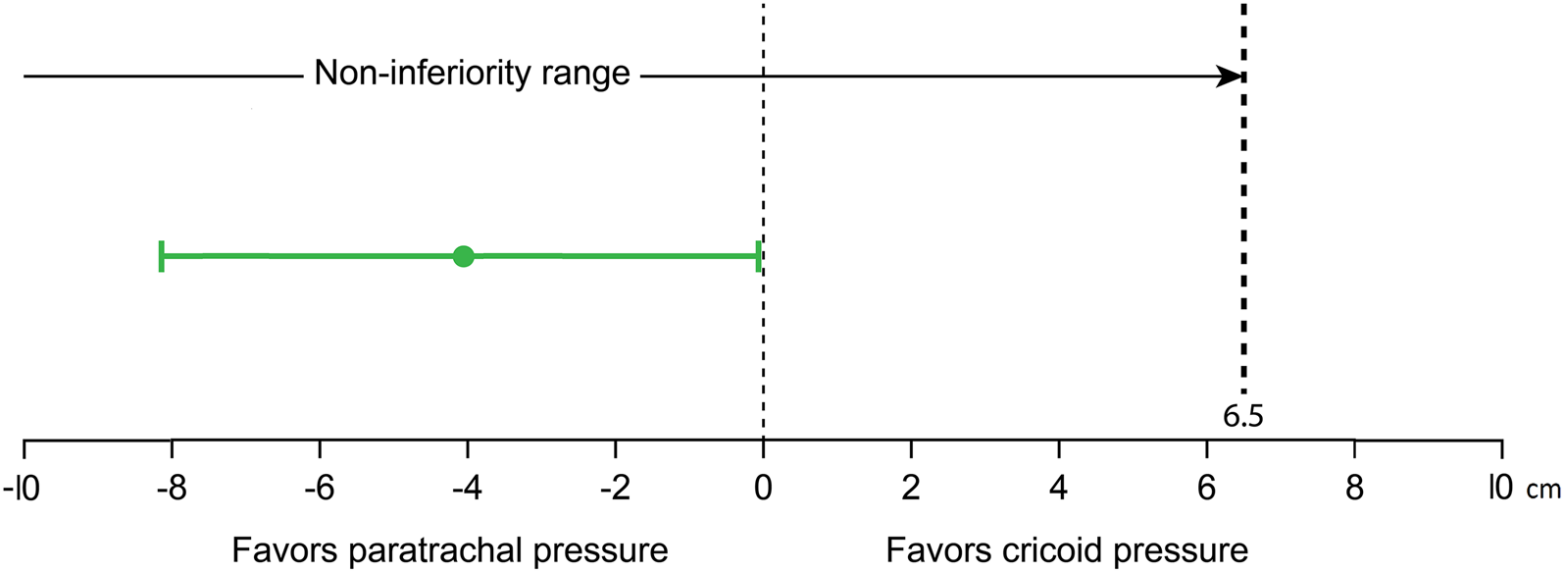
Difference in intubation time between paratracheal pressure group and cricoid pressure group. The green dot and plot indicate the mean difference and 95% CI. The noninferiority margin was set at 6.5 s.

Characteristics related to airway and intubation are shown in Table 2. There was no difference between the two groups in baseline examination to predict airway difficulty, including Mallampati class, thyromental distance, mouth opening, neck mobility grade, class of upper lip bite test, and El-Ganzouri Risk Index. The number of intubation attempts was comparable between the two groups. Noninferiority of the PP group compared to the CP group was proven because the upper limit of the 95% CI for the mean difference in intubation time (the PP group − the CP group) was within the non-inferiority margin of 6.5 s (Fig. 2). The mean difference in intubation time between the two groups was − 4.19 s (95% CI − 8.24 to − 0.15) (*p* < 0.001). The scores for glottic view at Pentax, including the Cormack–Lehane (C–L) grade and the percentage of glottis opening (POGO), and the intubation difficulty scale (IDS) were comparable between the two groups. However, the numeric rating scale (NRS) for tube advancement difficulty was significantly lower in the PP group than in the CP group (*p* = 0.02). Two patients (4.3%) in the PP group and 10 patients (22.2%) in the CP group needed rotation of the tube through 180 degrees to pass through the glottis (*p* = 0.01).

**Table 2:** Characteristics related to airway and tracheal intubation.

	PP group (n = 46)	CP group (n = 45)	*P*-value
Mallampati I/II/III/IV, n	24/19/3	22/20/3	0.95
Thyromental distance, cm	8.0 (6.5–9.0)	7.5 (7.0–8.3)	0.42
Mouth opening, cm	4.5 (4.0–5.0)	4.5 (4.0–5.0)	0.52
Neck mobility, normal n (%)	46 (100)	45 (100)	> 0.999
Upper lip bite grade (1/2/3), n	36/9/1	33/11/1	0.85
Intubation attempt (1/2/3), n	43/3/0	40/4/1	0.54
C–L grade (1/2a), n	41/5	36/9	0.23
POGO grade, %	100 (90–100)	100 (90–100)	0.23
El-Ganzouri risk index	2 (1–3)	2 (2–3)	0.17
Intubation difficulty scale	0 (0–0)	0 (0–1)	0.07
NRS for tube passage	0.5 (0–3.0)	3.0 (0–5.0)	0.02

*C–L* Cormack–Lehane, *POGO* percentage of glottis opening, *NRS* numeric rating scale.

Continuous variables are presented as median (25th to 75th quartile). Nominal variables are presented number or number (frequency).

**Figure 3:**
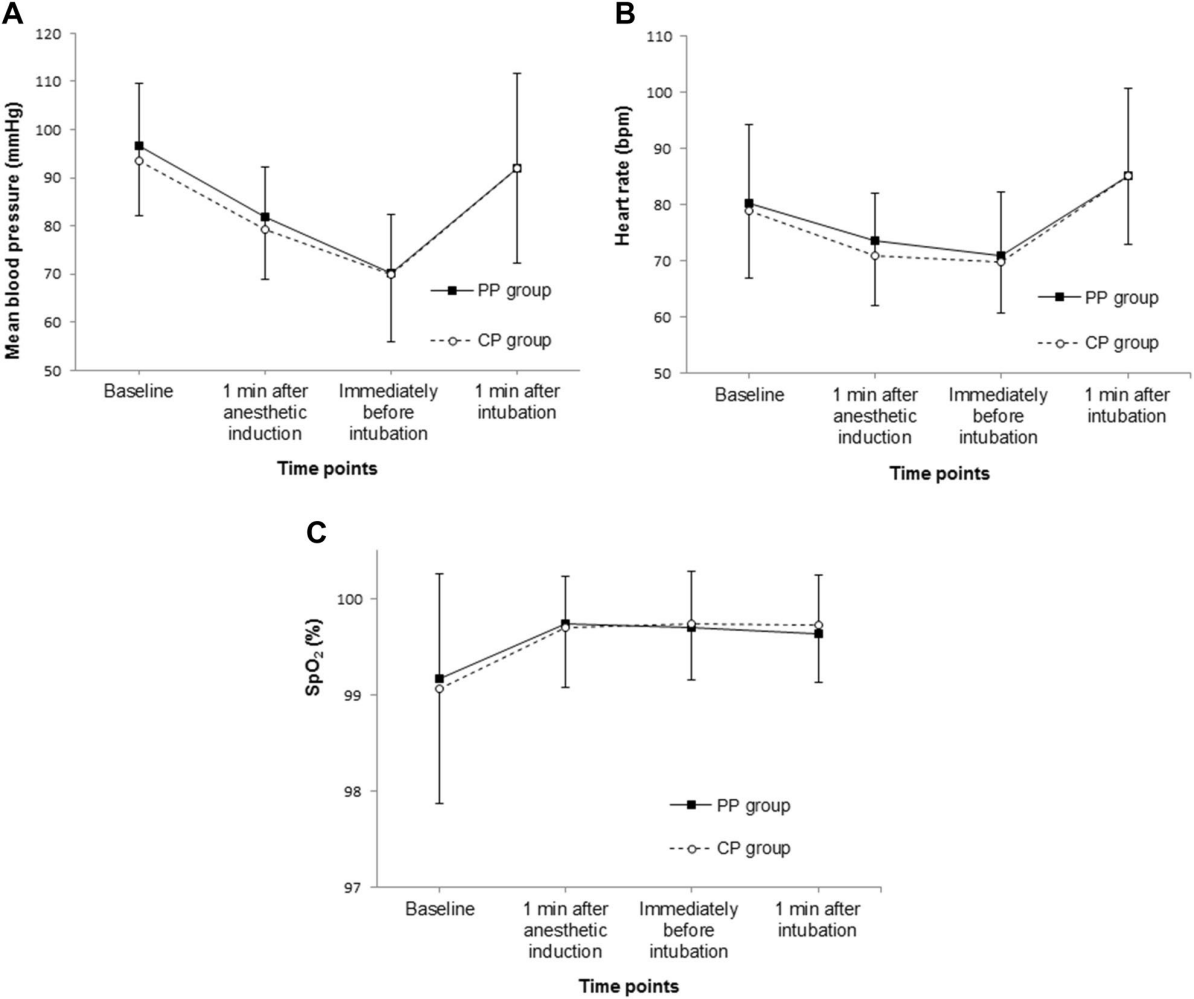
Changes of hemodynamics and saturation before and after intubation. *SpO*_*2*_ saturation via peripheral pulse oximetry, *PP group* paratracheal pressure group, *CP group* cricoid pressure group.

Changes of hemodynamics and saturation before and after intubation showed similar trends over time between the two groups (Fig. 3, all *p*s > 0.05). Similar trends over time were observed in the prevalence of hoarseness and pharyngeal pain at 1 h and 24 h postoperatively between the two groups (Table 3).

**Table 3:** Complications associated with intubation.

	PP group (n = 46)	CP group (n = 45)	*P*-value
Hoarseness 1 h	5 (10.9%)	11 (24.4%)	0.32
Hoarseness 24 h	2 (4.3%)	2 (4.4%)	
Sore throat 1 h	1.86 ± 1.63	2.09 ± 2.19	0.54
Sore throat 24 h	0.59 ± 1.00	0.57 ± 0.70	

Continuous variables are presented as mean ± SD. Nominal variables are presented number (frequency).

## Discussion

In this randomized, non-inferiority study, PP did not prolong the intubation time and did not worsen the glottic view, and furthermore improved tube advancement through the glottis compared to CP during intubation using Pentax. Hemodynamic changes during intubation and postoperative airway complications, including hoarseness and pharyngeal pain, were comparable between the two groups.

Komasawa et al. found that CP increased the intubation time and the difficulty of tube advancement^13^. Since PP can also cause tracheal deviation by causing pressure on the left side, and may interfere with tube advancement during intubation, we designed this study as a non-inferiority study comparing the differences between the CP and PP groups. In this study, PP did not prolong the intubation time compared to CP in intubation using Pentax. Rather, the intubation time tended to decrease in the PP group compared with the CP group [27 (25–34) s vs. 31 (27–40) s, *p* = 0.02]. In situations beyond safe apnea time, prolonged apnea time can lead to a rapid decline in arterial oxygen saturation and neurological damage, reaching rates of 3–8% per second, especially in critically ill, obese, pregnant, or pediatric patients^15,16^. Therefore, although the difference of 4 s is small, in critical circumstances even a small time reduction in intubation time will help reduce the hypoxic damage risk.

The trend towards a decrease in intubation time in the PP group compared to the CP group in this study may be associated with the ease of tube advancement. Considering the difference in NRS for tube advancement difficulty, CP seems to interfere with tube advancement more than PP in this study. The difficulty with tube advancement may be due to the difference in angle between the axis of tube advancement and the axis of the trachea during intubation using Pentax. Komasawa et al. have explained that CP may exacerbate the difference in angle by displacing the trachea downward^13^. In contrast, because PP does not cause direct pressure on the trachea, the change in angle in the PP group during intubation may be less than that in the CP group. The difficulty with tube advancement was resolved by rotating the tube through 180 degrees. Glottic view through the Pentax was clear in both groups (C–L grade ≤ 2a in all patients and median POGOs were 100 in both groups) and statistically comparable between the two groups.

Although there was a significant difference in tube advancement difficulty between the two groups, this did not lead to an increase in intubation attempts (success rate on the first attempt: 94% in the PP group vs. 89% in the CP group, *p* = 0.54), unlike the study by Komasawa et al.^13^. In our study, failure on the first attempt was caused by the failure of the Pentax blade to lift the epiglottis.

There has been controversy about the effectiveness of CP in studies based on anatomical structures. Some studies found deviation of the esophagus at the level of the cricoid cartilage on magnetic resonance imaging in 90.5% of subjects^17^, and CP was less effective in these cases^18^. In contrast, other studies revealed such lateral deviation did not reduce the effectiveness of CP because the esophagus posterior to the cricoid cartilage moved together with the larynx as a unit when CP was applied^19,20^. Further to the controversy regarding CP, Andruszkievicz et al., using ultrasound, reported that left paralaryngeal pressure at cricoid cartilage level effectively compressed the esophagus, whereas CP did not^21^. However, at this level, the esophagus could not be identified by ultrasound in 13% of patients. Gautier et al. modified the site of pressure to slightly lower, so as to identify the esophagus more consistently; they named this pressure PP^14^. In their study, left PP significantly reduced air entry into the gastric antrum during facemask ventilation, compared to CP. Therefore, we chose PP instead of paralaryngeal pressure to compress the esophagus. As a result, we identified the esophagus in all patients who were examined by ultrasound and confirmed that the esophagus was significantly compressed by 30 N of paratracheal pressure.

There were a few limitations to our study. Firstly, although a previous study revealed the efficacy of PP and CP in preventing air entry into the gastric antrum using ultrasound^14^, we did not evaluate whether the pressures actually prevented gas entry. Secondly, we underwent training to apply sustained pressure of 30 N; however, we cannot guarantee that this pressure was actually applied. Thirdly, the type of surgery may have influenced the evaluation of postoperative hoarseness and pharyngeal pain because we included patients undergoing otorhinolaryngology surgery. Lastly, we did not include the patients who anticipated difficult intubation and extreme obese patients. Therefore, our results cannot be applied to these patients.

In conclusion, PP did not prolong the intubation time and showed comparable intubation conditions compared to CP in intubation using Pentax. Therefore, PP may be a good alternative maneuver for prophylaxis in patients at risk of gastric regurgitation during intubation using Pentax.

## Methods

### Study population

This study was designed as a prospective, double-blind randomized, non-inferiority study. All methods were performed in accordance with the relevant guidelines and regulations. After approval by Ajou Hospital Institutional Review Board (AJIRB-MED-INT-19-340) and registering at ClinicalTrial.gov (NCT04135651, the date of first registration: 09/12/2019), written informed consent was obtained from all participants. Patients aged 19–70 years with American Society of Anesthesiologists physical status of 1 or 2 who underwent surgery under general anesthesia with intubation were included. Exclusion criteria were as follows: patients with loose teeth, those requiring rapid sequence intubation, where difficult intubation was anticipated (Mallampati grade 4 or mouth opening < 3 fingers), those with a history of gastro-esophageal reflux disease, body mass index more than 35 kg/m^2^.

### Randomization

Patients (*n* = 94) were randomized into one of the following two groups in a 1:1 ratio: PP group (*n* = 47) or CP group (*n* = 47) using a random sequence generator (http://www.random.org) by SKM. Group assignment was concealed using sealed and opaque envelopes. All outcome assessors were blinded to allocated group.

### Anesthesia and study protocol

Before the initiation of the study, JYK and HYK trained with a top-board weight scale (KS-514WT, Dretec, Kawaguchi, Japan) to apply sustained PP or CP of 30 N within 5 N error. The training was continued until 10 consecutive successes (30 ± 5 N) were achieved with a 13–6 MHz linear ultrasound probe, the thumb (to apply PP), and thumb and index fingers (to apply CP) of the right hand.

The patient arrived in the operating room without premedication. Standard monitoring, including peripheral pulse oximetry, electrocardiogram, noninvasive blood pressure, and bispectral index, was applied to the patient. The patient’s head was kept in a slight ‘‘sniffing’’ position, resting on a gel head donut, and the neck was supported by a 10 cm cotton roll. Induction of anesthesia was commenced with target-controlled mode using propofol (effect site concentration 5.0 mg/ml) and remifentanil (effect site concentration 4.0 ng/ml), and followed by rocuronium 0.8 m/kg. Thereafter, HYK opened the envelope to confirm the group allocation of the patient. While manual mask ventilation with 100% oxygen was applied for 5 min, the esophagus was examined using a 13–6 MHz linear ultrasound probe (SONIMAGE HS2, Konica Minolta Inc., Tokyo, Japan) between the left trachea and the sternocleidomastoid muscle just above the clavicle in the supine position without neck rotation by JYK. Immediately before intubation, the patient's neck was covered with an opaque cloth to keep the patient's group assignment blind to other investigators. Thereafter, HYK applied PP or CP with a force of 30 N. PP was applied to the space between the trachea and the sternocleidomastoid muscle just above the clavicle on the patient’s left side with the thumb of the right hand. CP was applied at cricoid cartilage level with the thumb and index fingers of the right hand. Intubation was attempted with Pentax by JHM, who had experience using Pentax at least 50 times after confirming the train-of-four ratio was zero with a train-of-four watch (TetraGraph, Senzime AB, Braintree, United Kingdom). The endotracheal tube had an inner diameter of 7.5 mm for males and 7.0 mm for females. PP or CP was released when the Pentax was removed from the patient’s mouth. During the intubation process, the attempt was terminated if the saturation of peripheral pulse oximetry dropped below 95%, or the intubation time was delayed beyond 60 s; mask ventilation was applied until oxygen saturation had increased to 100%. A maximum of three intubation attempts were allowed. Successful intubation was confirmed when end-tidal carbon dioxide was detected using capnography. If intubation failed after three attempts, failure of intubation was declared and standard intubation without obligatory pressure application was performed.

### Outcome measurements

In the pre-anesthetic room, the in-room anesthesiologist who did not know the patient’s group allocation measured a modified Mallampati class, thyromental distance, interincisor distance, a 3-graded neck mobility (normal, reduced, or fixed flexion or extension), and a class of upper lip bite test^22^. In addition, El-Ganzouri Risk Index was calculated for difficult airway prediction^23^.

Before intubation, the esophagus was examined and the axial and sagittal outer anteroposterior diameters of the esophagus were obtained with and without application of 30 N pressure using the ultrasound probe. After insertion of the Pentax into the patient’s mouth, the glottic view was evaluated using C–L grade^24^ and POGO^25^. During intubation, intubation time, defined as the time between insertion of the Pentax to confirmation of end-tidal carbon dioxide, was recorded. If there was more than one intubation attempt, the intubation time was calculated as the sum of the attempts. Intubation difficulty was evaluated using IDS. The IDS was the sum of the following seven variables: *N*1, number of intubation attempts; *N*2, number of operators; *N*3, number of alternative intubation techniques used; *N*4, glottic exposure by C–L grade; *N*5, force to lift equipment; *N*6, necessity for external laryngeal pressure; and *N*7, position of the vocal cords at intubation^26^. Of these seven variables, *N*6 was scored as zero if no additional force other than PP or CP of 30 N was applied. The difficulty of tube advancement through the glottis was evaluated through an 11-point NRS (0: extremely easy, 10: extremely difficult). Hemodynamics including mean blood pressure and heart rate, and saturation via peripheral pulse oximetry were collected at four time points: baseline, one minute after anesthetic induction, immediately before intubation, and 1 min after intubation. Postoperative hoarseness and pharyngeal pain were assessed by the attending nurse who questioned patients to give “yes or no” answers and the 11-point NRS (0: none, 10: the worst), respectively. These were assessed at 30 min after postanesthetic care unit arrival and 24 h after surgery.

### Statistical analysis

This study was planned as a non-inferiority design. The sample size was calculated using an online software (https://www.sealedenvelope.com/power/continuous-noninferior/). The sample size was calculated from intubation time, which was our primary outcome. The mean intubation time and SD were 43 ± 12 s in a previous study that applied CP in intubation using Pentax^13^. Assuming 15% (6.5 s) as the non-inferiority margin from the mean value in the previous study, 43 patients were required in each group with an alpha of 0.05 (one-tailed) and a power of 0.8. Considering a dropout rate of 10%, 47 patients per group were required.

Descriptive statistics were used to present patients’ demographics and characteristics related to airway and intubation. Categorical data were assessed using the chi-squared or Fisher’s exact tests and presented as numbers (frequency). Continuous data were assessed using independent *t* tests or Mann–Whitney *U* tests and presented as means ± SDs or medians (25th to 75th quartile). Repeated measure data were assessed using a generalized estimating equation model. Values of *p* < 0.05 were considered statistically significant.

Non-inferiority was assessed by the mean and 95% CI for the difference in intubation time between the two groups. If the upper limit of the 95% CI for the mean difference in intubation time (the PP group − the CP group) was within 6.5 s, we declared that Pentax intubation applying PP was not inferior to that applying CP. Statistics analyses were performed with SPSS (version 25.0, IBM Corporation, Armonk, NY, USA) and R (version 3.6.1).

## Data Availability

The datasets generated during and/or analyzed during the current study are available from the corresponding author on reasonable request.

## References

[1] Sellick BA (1961). Cricoid pressure to control regurgitation of stomach contents during induction of anaesthesia. Lancet (London, England).

[2] Fenton PM, Reynolds F (2009). Life-saving or ineffective? An observational study of the use of cricoid pressure and maternal outcome in an African setting. Int. J. Obstet. Anesth..

[3] Robinson JS, Thompson JM (1979). Fatal aspiration (Mendelson's) syndrome despite antacids and cricoid pressure. Lancet (London, England).

[4] Schwartz DE, Matthay MA, Cohen NH (1995). Death and other complications of emergency airway management in critically ill adults. A prospective investigation of 297 tracheal intubations. Anesthesiology.

[5] Haslam N, Parker L, Duggan JE (2005). Effect of cricoid pressure on the view at laryngoscopy. Anaesthesia.

[6] Hartsilver EL, Vanner RG (2000). Airway obstruction with cricoid pressure. Anaesthesia.

[7] Allman KG (1995). The effect of cricoid pressure application on airway patency. J. Clin. Anesth..

[8] Tournadre JP, Chassard D, Berrada KR, Boulétreau P (1997). Cricoid cartilage pressure decreases lower esophageal sphincter tone. Anesthesiology.

[9] Ralph SJ, Wareham CA (1991). Rupture of the oesophagus during cricoid pressure. Anaesthesia.

[10] Suzuki K, Kusunoki S, Tanigawa K, Shime N (2019). Comparison of three video laryngoscopes and direct laryngoscopy for emergency endotracheal intubation: A retrospective cohort study. BMJ Open.

[11] Bogdański Ł (2015). Simulated endotracheal intubation of a patient with cervical spine immobilization during resuscitation: A randomized comparison of the Pentax AWS, the Airtraq, and the McCoy Laryngoscopes. Am. J. Emerg. Med..

[12] Komasawa N, Kido H, Mihara R, Minami T (2017). Comparison of cricoid pressure effect between McGRATH(R) MAC and Pentax-AWS Airwayscope(R): A prospective randomized trials. Am. J. Emerg. Med..

[13] Komasawa N, Kido H, Miyazaki Y, Tatsumi S, Minami T (2016). Cricoid pressure impedes tracheal intubation with the Pentax-AWS Airwayscope(R): A prospective randomized trial. Br. J. Anaesth..

[14] Gautier N (2019). The effect of force applied to the left paratracheal oesophagus on air entry into the gastric antrum during positive-pressure ventilation using a facemask. Anaesthesia.

[15] Farmery AD, Roe PG (1996). A model to describe the rate of oxyhaemoglobin desaturation during apnoea. Br. J. Anaesth..

[16] Sands SA (2009). A model analysis of arterial oxygen desaturation during apnea in preterm infants. PLoS Comput. Biol..

[17] Smith KJ, Dobranowski J, Yip G, Dauphin A, Choi PT (2003). Cricoid pressure displaces the esophagus: An observational study using magnetic resonance imaging. Anesthesiology.

[18] Boet S (2012). Cricoid pressure provides incomplete esophageal occlusion associated with lateral deviation: A magnetic resonance imaging study. J. Emerg. Med..

[19] Rice MJ (2009). Cricoid pressure results in compression of the postcricoid hypopharynx: The esophageal position is irrelevant. Anesth. Analg..

[20] Zeidan AM (2014). The effectiveness of cricoid pressure for occluding the esophageal entrance in anesthetized and paralyzed patients: An experimental and observational glidescope study. Anesth. Analg..

[21] Andruszkiewicz P (2016). Ultrasound evaluation of the impact of cricoid pressure versus novel 'paralaryngeal pressure' on anteroposterior oesophageal diameter. Anaesthesia.

[22] Khan ZH, Kashfi A, Ebrahimkhani E (2003). A comparison of the upper lip bite test (a simple new technique) with modified Mallampati classification in predicting difficulty in endotracheal intubation: A prospective blinded study. Anesth. Analg..

[23] el-Ganzouri AR, McCarthy RJ, Tuman KJ, Tanck EN, Ivankovich AD (1996). Preoperative airway assessment: Predictive value of a multivariate risk index. Anesth. Analg..

[24] Yentis SM, Lee DJH (1998). Evaluation of an improved scoring system for the grading of direct laryngoscopy. Anaesthesia.

[25] Levitan RM, Ochroch EA, Kush S, Shofer FS, Hollander JE (1998). Assessment of airway visualization: Validation of the percentage of glottic opening (POGO) scale. Acad. Emerg. Med. Off. J. Soc. Acad. Emerg. Med.

[26] Adnet F (1997). The Intubation Difficulty Scale (IDS): Proposal and evaluation of a new score characterizing the complexity of endotracheal intubation. Anesthesiology.

